# Development and Validation of an App Rating System for Caregiver Financial Management Apps

**DOI:** 10.1177/30495334251379916

**Published:** 2025-09-24

**Authors:** Emma Cho, Mary D. Naylor, George Demiris, Sarah Stephens Winnay, Aysel Cibildak

**Affiliations:** 1 The University of Pennsylvania School of Nursing, PA, USA; 2 Archangels, PA, USA

**Keywords:** caregiving, financial, evaluation scale

## Abstract

**Background:**

This study aims to develop and evaluate a consumer-centered app rating system designed to guide employed caregivers in the selection of effective and user-friendly apps to manage financial stress.

**Methods:**

This study describes the development of a consumer-centered app rating system designed to guide employed caregivers in the selection of effective and user-friendly apps to manage financial stress and its psychometric evaluation using a sample of 100 US adults age 20 to 87 who were recruited from nine organizations through the Exhale respite grant website in February 2024.

**Results:**

Exploratory factor analysis indicated a two-factor structure comprised of user experience and financial management. The 13-item scale was psychometrically sound and demonstrated good internal reliability.

**Discussion:**

The development and evaluation of a scale to assess caregiver-specific financial management apps provides practical insights to improve the design of existing financial management apps and better support caregivers in their important and challenging financial role.

## Introduction

Approximately 6 in 10 employees are caring for family members or friends with serious health problems or disabilities (Feinberg & Skufca, 2020) and 60% of them are working full-time (AARP Family Caregiving, 2020). High levels of stress, reported by 4 in 10 working caregivers, are associated with negative short and long-term health consequences (Blue Cross Blue Shield Association, 2020; Haley et al., 2020). A major source of vulnerability for working caregivers is financial stress (Lerner, 2022). Nine in 10 caregivers report contributing their own income and savings to support their care recipient (financial contributors) or paying their care recipient’s bills, monitoring bank accounts, and handling bills (financial coordinators; Merrill Lynch & Age Wave, 2017). In addition, more than half of caregivers (53%) report that after 2 years of providing care, a family member or friend needs full financial support (Merrill Lynch & Age Wave, 2017).

In addition to personal sacrifices and increased family stress, one in four caregivers reported going into debt and reducing savings planned for their children’s education and retirement (Skufca & Rainville, 2021; Surya et al., n.d.). Hourly workers, women, and members of the Black, Hispanic, and LGBTQ communities reported greater strain. For those who leave the workforce or retire early (the majority of whom are women), the long-term financial impact is devastating (Merrill Lynch & Age Wave, 2014).

Data show that the proportion of workers who are caregivers and the demands associated with this role are expected to increase significantly in the near future. Recognizing the threat to recruitment, productivity, and retention posed by increased financial stress, some employers are responding by improving their employees’ access to benefits and resources (Bank of America, 2021).

One easily accessible resource that offers potential for promoting employee financial well-being is apps. Apps, computer software applications for any hardware platform (Phongtraychack & Dolgaya, 2018), are commonly specialized programs that can be downloaded by users on mobile devices. Apps can engage employees in meaningful financial planning, especially if the program anticipates their future role as caregivers (Shen et al., 2025). Importantly, apps can be used to support current caregivers in their roles as financial contributors and coordinators (Kelley et al., 2024). Financial planning and coordination apps, including apps specifically for caregivers, are ubiquitous (Brown et al., 2019). However, a significant challenge is that most apps are not free, and there is no reliable consumer-centered rating system to guide employers and employees in selecting apps that can improve financial well-being. As a result, caregivers are left to navigate a complex landscape of commercially available solutions and attempt to determine which solutions may be appropriate, trustworthy, and useful.

When it comes to rating systems for health-related apps broadly, the most rigorously developed and widely used rating system is the Mobile App Rating System (MARS; Stoyanov et al., 2015), MARS was developed by a panel of experts to evaluate mHealth apps. It includes basic categories such as engagement, functionality, esthetics, information quality, and overall satisfaction. However, the scale was designed to help researchers and healthcare professionals assess the quality of mHealth apps. Findings from one study using the MARS subscales to identify consumer needs and preferences highlighted that, while the scale provides a general guide to attributes that may be important to consumers, more work is needed to facilitate reliable consumer-centered evaluation (Choi et al., 2018). Therefore, the purpose of this research is to develop and test a consumer-centered app rating system designed specifically to guide employed caregivers in the selection of effective and user-friendly apps to manage financial stress.

## Method

### Study Overview

A multi-assessment model, incorporating both qualitative (e.g., focus groups) and quantitative methods (e.g., exploratory factor analysis), was used to develop a consumer-centric app rating system for financial apps to support caregivers. The specific assessments and procedures are described in detail in the methods section.

The study was approved by the Institutional Review Board of the University of Pennsylvania. Participation in the qualitative and quantitative assessments was voluntary, anonymous, and confidential, and participants were informed of the purpose and procedures of the study and signed written consent forms. Data were stored on a password-protected laptop computer and only the researchers had access to the data. In the sections below, the methods and findings from each of the study phases are described.

#### Phase I-Qualitative Phase/Development of Application Rating Systems (APS)

An extensive literature review was conducted to identify important themes for caregivers in managing key sources of financial stress. Previous studies have emphasized the financial stress of caregiving tasks, including medication management (Maidment et al., 2017) and lost income from reduced work hours (Reinhard et al., 2015). Caregivers also report difficulty accessing trustworthy financial information and making complex decisions under stress (Fan et al., 2023). The following themes also were informed by data related to the sources and impact of financial stress provided by team members as well as the MARS rating scale:

- App classification, confidentiality, security, registration, community, affiliation
- Esthetics, graphics, layout, visual appeal
- Engagement, entertainment, customization, interactivity, fit to target group
- Functionality, performance, navigation, gestural design, ease of use
- Information, quality, quantity, visual information, credibility, goals, description

Subjective quality, worth recommending, stimulates repeat use, overall satisfaction rating. Using an iterative design approach, we conducted three focus groups with employees who also are caregivers to elicit their feedback about priorities and expectations regarding managing sources of financial stress as well as their overall attitudes toward app use. Feedback from each focus group informed modifications to the list of themes prior to the next session. Working caregivers for the first two groups were recruited from aging and healthcare organizations located in western New York and Detroit and large hospital networks in Buffalo and Detroit. Participants had to be at least 18 years of age, employed full time, and currently serving as caregivers. These initial sessions were led by a qualitative researcher who used the themes noted above and subsequently refined to guide each conversation. Each of these 90- min sessions were conducted via zoom. Each participant who completed these focus groups received a $50 stipend. The third focus group session was conducted in person with participants. Participants for the in-person group were employees of a large non-profit that provides housing, health and social services to marginalized adults in the Philadelphia area who also were caregivers; these participants also received a $50 stipend. All sessions were audio-recorded and transcribed prior to content analysis.

The recorded transcripts were subjected to content analysis based on the Technology Acceptance Model (TAM; Davis, 1989) to predict respondents’ intentions to use technology, using NVivo qualitative data analysis software. The codes obtained from the analysis provided the team with the information they needed to understand users’ perceptions of the usefulness and ease of use of the features included in the rating system application, and to shape changes before the next usability cycle.

#### Phase II-Quantitative Phase/Psychometric Properties of the APS

For the second phase of this study, a survey that included the 17 items identified in Phase I was administered to a larger group of working caregivers with the goal of assessing construct validity using exploratory factor analysis (EFA). Currently employed caregivers over the age of 18 were eligible to complete the questionnaire online. Participants were recruited from nine organizations through the Exhale respite grant website (https://tpi.org/exhale-the-family-caregiver-initiative/) in February 2024. A total of 133 participants were recruited, with 100 participants completing all survey questions for a 75% response rate. Once recruited, participants who completed the survey were paid a $100 honorarium in the form of a gift card.

The survey was comprised of two parts. Part I allowed participants to rate the 17 items using a 5-point Likert scale based on what they deemed most important to them in selecting and using financial apps designed to support caregivers (Table 1). The survey has a Flesch Reading Ease of 62.5 and a Flesch-Kincaid Grade Level of 7.3. Participants were prompted to select an app they were currently using for managing finances, and to complete the scale for this specific app. Part II included three open-ended questions designed to elicit feedback on features participants would like to see in a rating system. Additionally, the Caregiving Intensity Index (CII) and basic demographic questions were included in the survey to explore potential correlations between CII scores, sociodemographic factors and the app rating system. The CII is a tool created by Rebel Health d/b/a ARCHANGELS available via the archangels.me website to all types of caregivers, including those caring for someone with dementia. States, employers, health systems, and other organizations contract with ARCHANGELS to provide the CII at a population level to support caregivers by providing them with an Intensity Score and their individual drivers and buffers and connecting them with resources while also providing aggregate reporting to the state/organization to guide policy and support investments.

**Table 1.** App Rating System Items Resulted from Qualitative Assessment (Phase I).

Survey Question	Strongly disagree	Disagree	Neutral	Agree	Strongly agree
1. The app is easy to find in an app store.	1	2	3	4	5
2. The app description in the store provided useful information about what it does.	1	2	3	4	5
3. The app is easy to use.	1	2	3	4	5
*4. The app allows me to tailor the way I use it based on my preferences.*	1	2	3	4	5
5. It is easy to learn how to use this app.	1	2	3	4	5
*6. The app is visually appealing.*	1	2	3	4	5
7. The information included in the app is accurate.	1	2	3	4	5
8. The app content is easy to understand.	1	2	3	4	5
9. The amount of information provided in this app is sufficient.	1	2	3	4	5
*10. The app provides information about its sources.*	1	2	3	4	5
11. I would recommend the app to people who may benefit from it.	1	2	3	4	5
*12. I feel any personal information or data I entered is secure and protected.*	1	2	3	4	5
13. The app helps me understand how to manage financial expenses.	1	2	3	4	5
14. This app makes me confident about my future financial security.	1	2	3	4	5
15. This app helps me resolve my financial stress.	1	2	3	4	5
16. The app provides me with the tools to have financial conversations with others involved in recipient’s care.	1	2	3	4	5
17. This app helps me understand how my finances contribute to my overall stress.	1	2	3	4	5

*Note.* Questions in *italics* are excluded after factor analysis.

Using SPSS, exploratory factor analysis was performed on the 17 survey items to assess the factorial validity of the scale, after first confirming that the data were suitable for factor analysis. Principal component analysis (PCA) was then used to extract the factors, followed by oblique rotation of the factors using oblimin rotation (δ = 0). The number of factors to be retained was guided by Kaiser’s criterion (eigenvalues greater than 1), inspection of the scree plot, and the interpretability of the items. Cronbach’s alpha was used to assess the internal consistency of the total scale and the subscales identified by PCA. Pearson correlation was used to explore the correlation between CII, app rating system, and demographic factors.

## Result

### Phase I

In the first focus group, 10 working caregivers (six female, four male) discussed expectations around financial apps designed to support caregivers and how individuals go about selecting and using apps. Based on the findings from this group, we expanded the list of themes and conducted a second online focus session with another eight working caregivers (five female, three male). Following the second session, we further refined the rating system and created an online version of the rating system that was available as a scale on an iPad. The third focus group session was conducted in person with 12 participants (eight female, four male) who had an opportunity to discuss both the items on this scale as well as the potential utility and usability of an app rating system that could be used on their desktop or smartphone. Participants emphasized the need for an app to be easy top use but also trustworthy and reliable. Emphasis was placed on knowing how data are collected, stored and whether one’s own personal data are shared with third parties. These themes were embedded into the resulting scale that included 17 items as described earlier (see Table 1), which was used for further testing during Phase II.

Guided by participants’ recommendations, considerable effort was put into describing the items on the rating scale in a clear, readable, and understandable way using simple language. The initial draft of the survey was developed with 25 items using a 5-point Likert scale (1 = strongly disagree to 5 = strongly agree). After analysis of findings from the three focus group sessions, the total number of items was reduced to 19. To address face validity, the survey was presented to an advisory group of design experts, and industry leaders in care, finance, and applications, and through an iterative process that included rewording and merging of some items, the version of the measurement tool for Phase I, presented in Table 1, and comprising 17 items was finalized.

### Phase II

The majority of the 100 participants in Phase II were female (69%), 30% were male, and 1% did not identify their gender. The mean age of the participants was 52.95 ± 13.9 years (range: 20–87). The majority of participants were white (82%), 9% were black, 3% were Asian, 3% were more than one race, and 3% did not identify their race (see Table 2).

**Table 2.** Demographic of the Study Population.

Demographic	*N* (%)	Mean (*SD*)
Age		52.95 (13.9)
Gender		
Male	30 (30)	
Female	69 (69)	
No answer	1 (1)	
Race		
White	82 (82)	
Black or African American	9 (9)	
Asian	3 (3)	
More than one race	3 (3)	
Unknown/not reported	3 (3)	

#### The Questionnaire as a Whole

The KMO measure of sampling adequacy was 0.886 and significance for Bartlett’s test of sphericity reached statistical significance (*χ*^2^ = 1141.649, *p* < .001), which shows that these items are factorable. The communalities of all the items were above 0.30 and the three factors were found to have eigenvalues greater than 1, accounting for 66.8% of total variance. The Cronbach’s alpha (α) value was .923 indicating that the internal consistency between items is very high.

#### Oblimin Rotation Method

The oblimin rotation method was used to identify meaningful components of the questionnaire. The four items 4 (The app allows me to tailor the way I use it based on my preferences), 6 (The app is visually appealing), 10 (The app provides information about its sources), and 12 (I feel any personal information or data I entered is secure and protected) did not contribute meaningfully to the components due to cross loading of 0.40 or more and interpretability issues (Taherdoost et al., 2014). To obtain better validated results, these four items were removed from the questionnaire upon completion of the factor analysis and tests were re-run on the retained items. In Table 1, the four items removed are in italics. The 13 items remaining comprise the final set of items for the app rating system.

The results of the factor analysis of the 13 remaining items showed that the KMO measure of sampling adequacy was 0.881 and the *p*-value of Bartlett’s test of sphericity reached statistical significance (*p* < .001), indicating that these items are factorable, and the data are very suitable for factor analysis. A total of two factors were found to have eigen values greater than 1, accounting for 67.8% of total variance. The communalities of the total items were greater than 0.30, indicating that Items 1, 2, 3, 5, 7, 8, 9, and 11 fit into Component 1, and Items 13, 14, 15, 16, and 17 fit into Component 2 (see Table 1).

Component 1 was labeled user experience. There were eight items that loaded highly on this factor, with loadings ranging from 0.61 to 0.88. The top three items within this factor were “The app content is easy to understand” (loading of 0.88), “The app is easy to understand” (0.85), and “The app description in the store provided useful information about what it does” (0.81).

The second component, financial management, contained five items with factor loadings ranging from 0.80 to 0.94. The top two items this component were “The app helps me understand how to manage financial expenses” (loading of 0.94) and “This app makes me feel confident about my future financial security” (loading of 0.87). Cronbach’s alpha reliability coefficients were calculated for each of the components identified from the PCA (Table 3). Both components had Cronbach alpha values greater than 0.7, indicating satisfactory internal consistency.

**Table 3.** Internal Consistency and Reliability (Subgroup).

Component	Cronbach’s alpha
Factor 1	.897
Factor 2	.920

#### Correlations Between CII, App Rating System, and Demographic Factors

Potential correlations between demographic factors, CII, and app rating system were explored. Older age and female participants reported significantly higher average ratings on aspects of financial management compared to their male counterparts (*p* < .05). There was no statistically significant relationship found between CII scores and the app rating system indicating the potential usefulness of an app rating systems for caregivers at all levels of intensity.

## Discussion

The purpose of the study was to develop and validate a rating system to assist employed caregivers in selecting apps designed to help them manage their roles as financial contributors and financial coordinators. To the best of our knowledge this is the first effort to fully engage consumers (i.e., employed caregivers) in both the design and testing of such a system. An app rating system that emerged from qualitative and quantitative assessments is comprised of 13 items were identified through psychometric analysis to include two factors: user experience and financial management.

In this study, the Technology Assessment Model (TAM) provided a robust foundation for evaluating financial apps by focusing on critical aspects that influence caregiver acceptance and use of these tools. By employing TAM-based content analysis, we ensured that our developed app rating system accurately reflected the factors that drive user adoption, such as perceived ease of use and perceived usefulness (Davis, 1989). This approach not only highlights the relevance of TAM in the context of financial apps but also underscores its utility in creating a comprehensive evaluation framework that addresses the specific needs and preferences of caregivers.

The user experience questions assess whether the app is easy to understand, accessible, accurate, and provides sufficient information. It also measures caregiver satisfaction and willingness to recommend the app to others. Boots et al. (2014) found that caregivers want supportive interventions for caregiving activities to include customized caregiving resources and support for those with low technology literacy. In addition, Ghahramani and Wang (2021) reported that caregivers increased adoption when the apps they used were convenient and helped them find useful resources and solutions. These findings highlight the importance of a positive user experience in app adoption and suggest that our evaluation system can provide predictive insights into financial management app adoption by identifying these caregiver preferences. As such, the system can help identify areas for improvement, such as intuitive navigation, personalized information, and minimizing barriers to enrollment, to significantly improve the caregiver experience significantly.

Questions about financial management include whether the app helps caregivers reduce financial stress and helps them understand how to manage their finances. A survey of 170 caregivers in Philadelphia found that one of the top concerns of caregivers was information or resources about financial support/assistance, and they also expressed interest in ways to improve their own emotional health and well-being (Health Union, 2022). These findings suggest that the app development system developed in this study may address an important gap in efforts to address caregiver financial stressors. In addition, the development of the app development system with a caregiver-centered design, including a “what matters to caregivers” perspective, suggests that we can better understand caregivers’ perspectives (Supplemental File).

Additionally, our results showed potential correlations between socio-demographic factors and the app rating system. Specifically, older age and being female were significantly positively correlated with the financial management aspects compared to their younger age group and male counterparts. These findings suggest that older users and females may have different needs or preferences that influence their financial management skills. Understanding these demographic differences is critical to developing more tailored and effective financial management apps for different user groups.

The validated app rating tool for caregiver financial management has multiple potential applications in clinical and community settings. Healthcare professionals, including social workers, case managers, and interdisciplinary geriatric care teams, can use the tool to recommend mobile applications that align with caregivers’ financial needs and digital literacy levels which allows for more personalized and effective support (Kelley et al., 2024). At the organizational level, public-facing entities, including government agencies (e.g., the administration for community living), nonprofit caregiver support organizations, and digital health repositories could serve as primary implementation bodies. State and federal agencies, for example, could use the tool to assess pilot programs integrating mobile financial management apps and inform policy decisions and reimbursement models tied to caregiver outcomes (Goodman, 1988). Additionally, app developers focusing on aging, caregiving, and financial wellness could incorporate the tool into their quality improvement processes. These diverse implementation pathways support the tool’s utility in guiding individual caregiver decision-making, enhancing clinical practice, and informing policy and program development aimed at supporting caregivers of older adults.

Despite its strengths, the study has limitations. Participants were predominantly White and recruited only from the eastern United States, which limits the generalizability of the findings. Future research should include a larger and more diverse sample to validate the effectiveness of the system across different racial and cultural backgrounds. In addition, further research is needed to examine the long-term effects of app use on caregivers’ financial stress and management skills. Examining other demographic subgroups and conducting longitudinal studies would deepen our understanding of how a rating system designed to assist caregivers in selecting apps can contribute to improvement in caregivers’ financial management.

## Conclusion

In conclusion, this study contributes a valuable evaluation framework for assessing caregiver-specific financial management apps. Our findings reinforce the critical need for apps that address the practical challenges caregivers face and suggest that app developers who focus on user-friendly and tailored solutions will see higher adoption rates. Ultimately, this work provides practical insights that can lead to improved app design and better support for caregivers in their critical and challenging financial role.

## Supplemental Material

sj-docx-1-ggm-10.1177_30495334251379916 – Supplemental material for Development and Validation of an App Rating System for Caregiver Financial Management Apps Supplemental material, sj-docx-1-ggm-10.1177_30495334251379916 for Development and Validation of an App Rating System for Caregiver Financial Management Apps by Emma Cho, Mary D. Naylor, George Demiris, Sarah Stephens Winnay and Aysel Cibildak in Sage Open Aging
