# Supplementary material for: Development and Validation of an App Rating System for Caregiver Financial Management Apps
Source: Sage Open Aging. 2025 Sep 24;11:30495334251379916. doi: 10.1177/30495334251379916 (PMC12461043; doi:10.1177/30495334251379916)
Supplement: sj-docx-1-ggm-10.1177_30495334251379916 – Supplemental material for Development and Validation of an App Rating System for Caregiver Financial Management Apps [file sj-docx-1-ggm-10.1177_30495334251379916.docx]

**App Rating System for Caregiver Financial Management Apps**

| **User experience** | | | | | |
| --- | --- | --- | --- | --- | --- |
|  | Strongly disagree | Disagree | Neutral | Agree | Strongly agree |
| 1. The app is easy to find in an app store. | 0 | 1 | 2 | 3 | 4 |
| 2. The app description in the store provided useful information about what it does. | 0 | 1 | 2 | 3 | 4 |
| 3. The app is easy to use. | 0 | 1 | 2 | 3 | 4 |
| 4. It is easy to learn how to use this app. | 0 | 1 | 2 | 3 | 4 |
| 5. The information included in the app is accurate | 0 | 1 | 2 | 3 | 4 |
| 6. The app content is easy to understand. | 0 | 1 | 2 | 3 | 4 |
| 7. The amount of information provided in this app is sufficient. | 0 | 1 | 2 | 3 | 4 |
| 8. I would recommend the app to people who may benefit from it. | 0 | 1 | 2 | 3 | 4 |
| **Financial management** | | | | | |
|  | Strongly disagree | Disagree | Neutral | Agree | Strongly agree |
| 1. The app helps me understand how to manage financial expenses. | 0 | 1 | 2 | 3 | 4 |
| 2. This app makes me feel confident about my future financial security. | 0 | 1 | 2 | 3 | 4 |
| 3. This app helps me resolve my financial stress. | 0 | 1 | 2 | 3 | 4 |
| 4. The app provides me with the tools to have financial conversations with others involved in recipient’s care. | 0 | 1 | 2 | 3 | 4 |
| 5. This app helps me understand how my finances contribute to my overall stress. | 0 | 1 | 2 | 3 | 4 |

SCORING: possible range of scores is 0 to 52, with higher scores indicating the greater overall quality and caregiver-perceived usefulness of the financial management app.
